# Supplementary figures and images for: Proanthocyanidins in seed coat tegmen and endospermic cap inhibit seed germination in Sapium sebiferum
Source: PeerJ. 2018 Apr 26;6:e4690. doi: 10.7717/peerj.4690 (PMC5924686; doi:10.7717/peerj.4690)

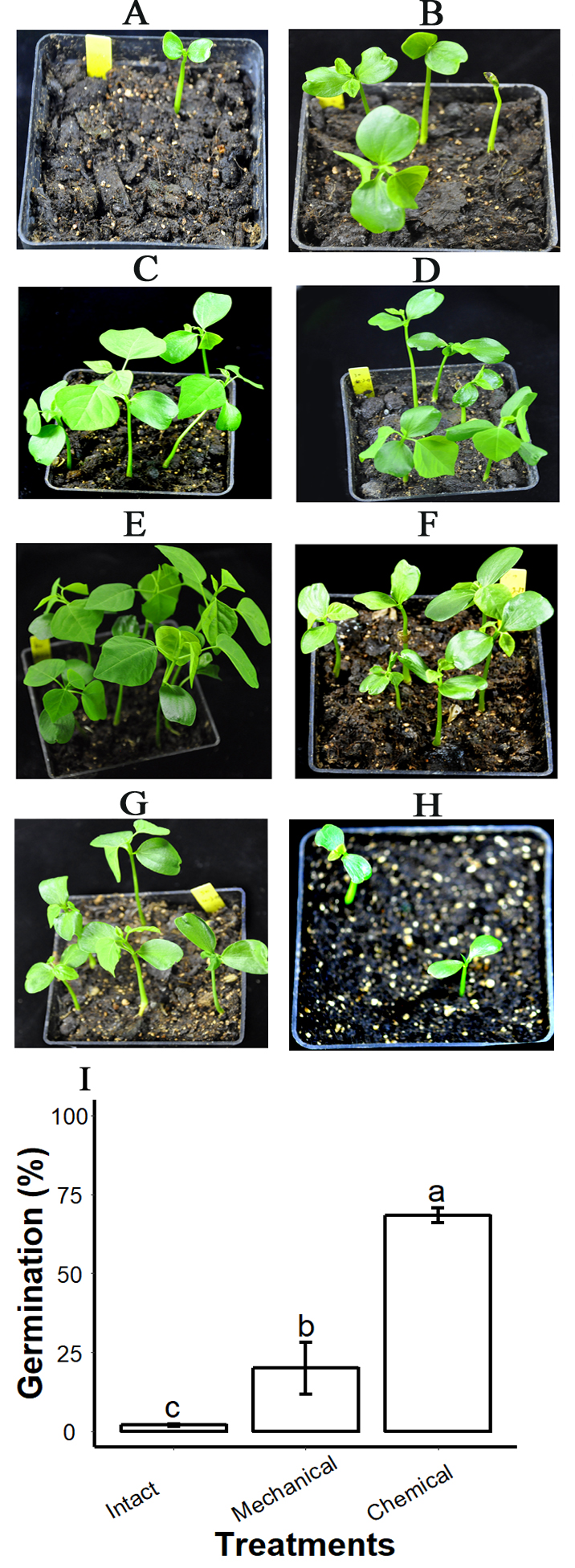

Supplement: Supplemental Information 1 — A, B, C, D, E, F and G are 0-(control), 10-, 20-, 30-, 40-, 50- and 60 minutes incubation with concentrated sulfuric acid respectively. H, mechanical scarification effect on seed germination. Seeds of every treatment were sown in each 10×10 cm pot with 3 replicates. Photographs were taken after one month of seed sowing. I, graphical comparisons of seed germination of intact seed, mechanical and sulfuric acid scarified seeds. Data were collected 30 days after seed sowing. Data shown are means±sd (n=3). Means with different letters are significantly different at P < 0.05 using Tuckey’s HSD post hoc test. The photographs were taken by Shah Faheem Afzal and Jun Ni. [file peerj-06-4690-s001.jpg]

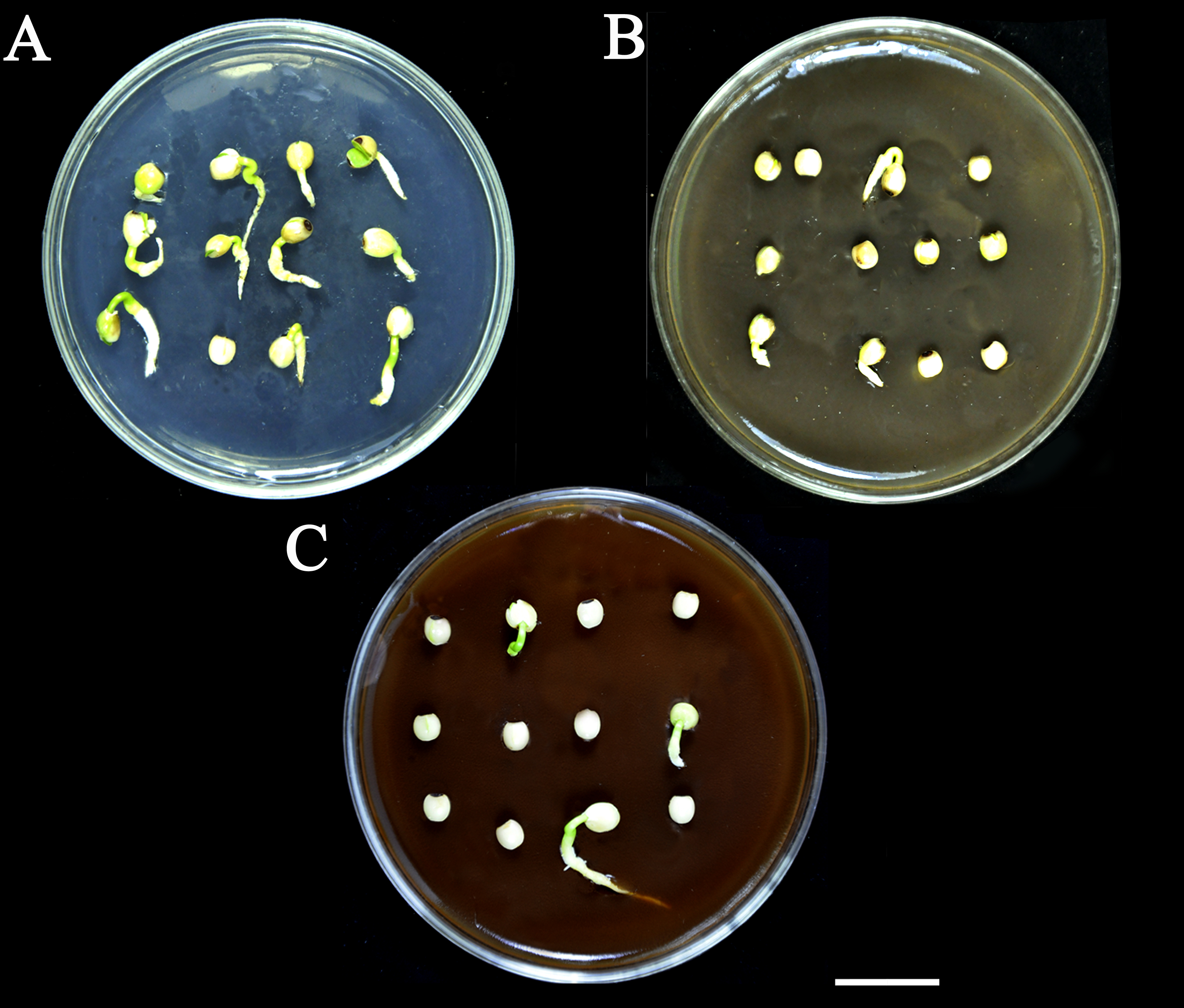

Supplement: Supplemental Information 2 — A, 0.5×MS (control). B, 0.3% SCE+0.5×MS. C, 0.1% PAs+0.5×MS. For each treatment, twelve seeds were sown in a 9 diameter cm Petri plate separately. All treatments were replicated 5 times. The photographs were taken on the 7th day of imbibition by Shah Faheem Afzal. Bar = 2cm. [file peerj-06-4690-s002.jpg]

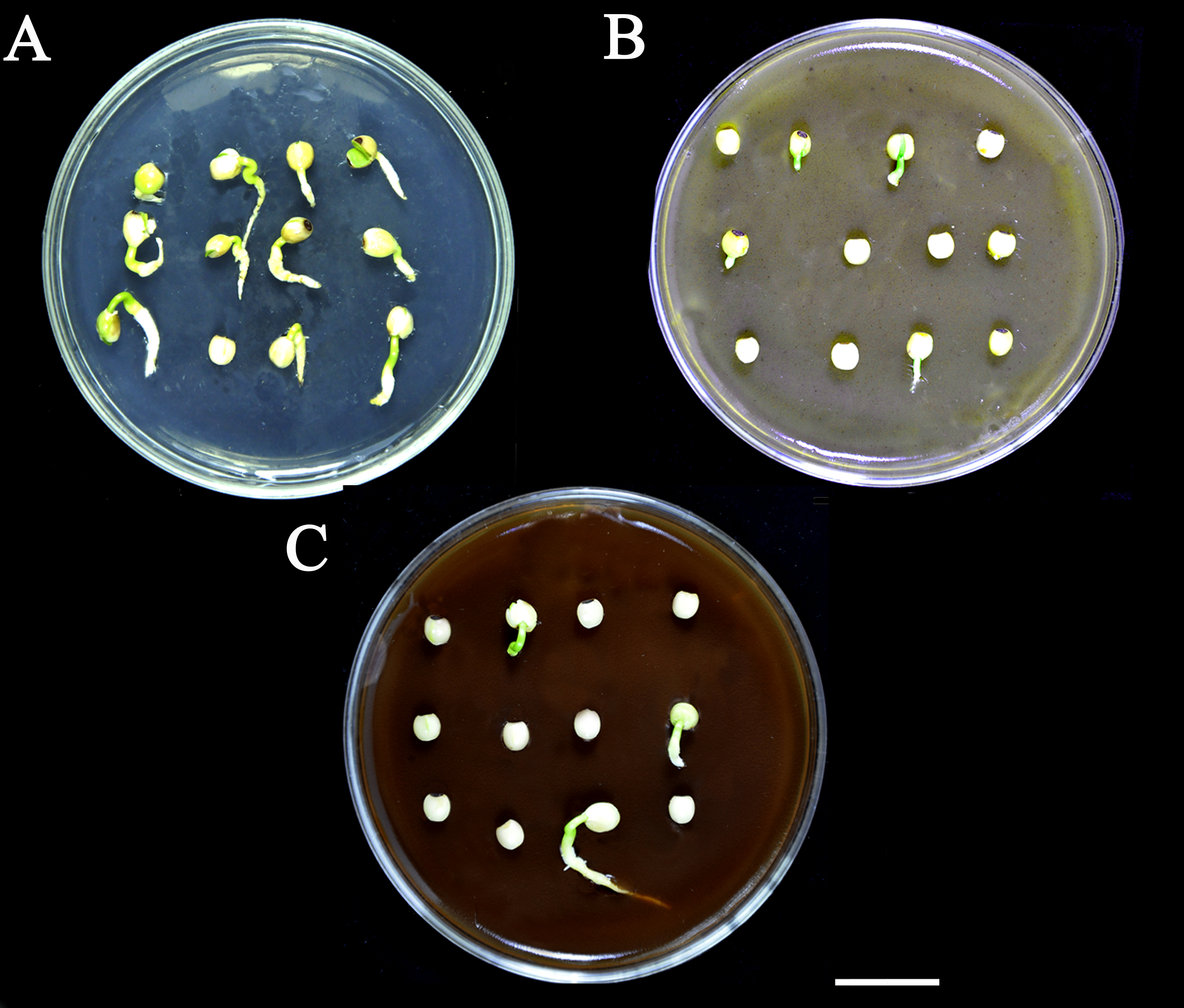

Supplement: Supplemental Information 3 — A, control (sterile water). B, 50 μΜ GA3. C, 50 μΜ NDGA. D, 20 mM H2O2 and E, 0.4% KNO3 priming overnight at room temperature and the primed seed of all treatments were grown separately on 0.3% SCE+0.5×MS in 9 cm Petri plates (12 seeds per plate) for 7 days. These photographs were taken on the 7th day of imbibition. Bars = 1 cm. The photographs were taken by Shah Faheem Afzal. [file peerj-06-4690-s003.jpg]
