# Supplementary material for: Proanthocyanidins in seed coat tegmen and endospermic cap inhibit seed germination in Sapium sebiferum
Source: PeerJ. 2018 Apr 26;6:e4690. doi: 10.7717/peerj.4690 (PMC5924686; doi:10.7717/peerj.4690)
Supplement: Supplemental Information 4 — The full sequences of all genes were obtained by local blasting Arabidopsis amino-acids sequence in blast-2.2.31. A local-blast library was built by S. sebiferum flower-bud transcriptome (Accession: SRX656554, https://www.ncbi.nlm.nih.gov/sra/SRX656554). The CDS regions of genes were translated by https://web.expasy.org/translate/. [file peerj-06-4690-s004.docx]

**Supplementary Data 1 Full length genes sequences with translated CDS**

***SsDOG1***

ccttggagtgagcaaagcccatttccgggaattttttgaagaaaatgattcaata atggcaaccaaggatgatcaaaagaatgcagaaagatgtttccttgaatggatgcaaatt

M A T K D D Q K N A E R C F L E W M Q I

caagaagctgatcttgcagagctactccaaaccctaagcctaaccgaaaaatctcaagat

Q E A D L A E L L Q T L S L T E K S Q D

ttgctcgcccaacttgcagagaagagcgtcaagcatttccaagattacatggagaggcga

L L A Q L A E K S V K H F Q D Y M E R R

attcaacatgcccacaacgatgcttcagcctattttgcaccgacatggaattccaccttg

I Q H A H N D A S A Y F A P T W N S T L

gagaattcattgatgtggttgggaggctgcaggccctccatttccatcagactcttatac

E N S L M W L G G C R P S I S I R L L Y

gcactctgtggctcagaggtcgaatcccatctcgcagaatacattcgaggtgtaagaaca

A L C G S E V E S H L A E Y I R G V R T

ggtaatctcggggatctttcagcagcacaactcaattcgatcaacgaattgcaccgcaaa

G N L G D L S A A Q L N S I N E L H R K

actgttaagcaagaggacaaattgacaacccaattggctggtttgcaagagaatatggct

T V K Q E D K L T T Q L A G L Q E N M A

gatgagcccatctcagggatcgcgaaaggcctaagccatgccggtgaagttaacaaggag

D E P I S G I A K G L S H A G E V N K E

gtggacagggcacttcaaaaccacgaaaattccatgcttcgtatgctgcaggaggctgat

V D R A L Q N H E N S M L R M L Q E A D

aatttacgcctaagcacgcttaaggagttagttagaatattaacccca

N L R L S T L K E L V R I L T P *

tcaggcagtagagtatctagctactgccaaaaagcttcacctctccttacgtcaatggggtaaaagaagggatcttatgcacggcagac

***SsNCED6***

gcacccacaggtggacaccacttgtttgatggtgatggt

atgaagagagatcatcaagaaagtgctggtggttgtggtgcgggcaaaggggagccctct

M K R D H Q E S A G G C G A G K G E P S

tctatggcgagaggaaaaatttgggaagaagaacaagatggtgcaggtatggatgaattg

S M A R G K I W E E E Q D G A G M D E L

cttgctgtcttgggttacaaggttcgatcttctgatatggcagatgtagctcagaagctt

L A V L G Y K V R S S D M A D V A Q K L

gaacagcttgaaatggtgatgggtactgctcaagaagatgggatttcacagctttctgat

E Q L E M V M G T A Q E D G I S Q L S D

accgtacattataatccctctgatctctctggttgggttcagagtatgctctctgagctt

T V H Y N P S D L S G W V Q S M L S E L

aatccagccactggacttgatgattctgtttttggttcttctgaaattagtttctctaat

N P A T G L D D S V F G S S E I S F S N

cctcaatcgcaccaatcgggtcttttcaatgatgattcggaatatgatctccgagcaatt

P Q S H Q S G L F N D D S E Y D L R A I

cccggtgtggtgggttacccacctgaggaaaatagcaggaagaaaatgaaaacctctatg

P G V V G Y P P E E N S R K K M K T S M

gggtctgatacagctgattcgaatccgaaaactccttcattagcttcaacttctggctcg

G S D T A D S N P K T P S L A S T S G S

ccgtcgcgagcggtggtgctaatcgactcgcaggagaccggagtccgcctagtgcatacg

P S R A V V L I D S Q E T G V R L V H T

ttattagcttgtgctgaagcagttcagcaagacaatttaaagctcgcagaggcactcgtc

L L A C A E A V Q Q D N L K L A E A L V

aaacacattggcttacttgctgcatctcaagccagtgctatgaggaaagtggcgacttac

K H I G L L A A S Q A S A M R K V A T Y

ttcgccgaagctttagctcgtcgaatttacaaaatctaccctcaagaatccctcctcgat

F A E A L A R R I Y K I Y P Q E S L L D

ccttcttattccgatactttagagatgcatttttacgagacttgcccttatctcaaattc

P S Y S D T L E M H F Y E T C P Y L K F

gcccattttactgctaatcaagccattcttgaagcttttggtactgcaaacagagttcat

A H F T A N Q A I L E A F G T A N R V H

gtcattgatttcggtctcaaacagggaatgcagtggccggccctaatgcaagctctcgct

V I D F G L K Q G M Q W P A L M Q A L A

ttgcgtcctggtggtcctcccactttccggttaaccggaatcggccctccgcagcccaat

L R P G G P P T F R L T G I G P P Q P N

aacaccgatactttgcagcaggtggggtggaagctagctcagttagctgaaactattggc

N T D T L Q Q V G W K L A Q L A E T I G

gtggaattcgaattccgcggtttcgtagctaatagtttagcagatcttcaaccggaaatt

V E F E F R G F V A N S L A D L Q P E I

ctggacctccgctccccggaggtggagacggtggttgtcaactccgttttcgagctccac

L D L R S P E V E T V V V N S V F E L H

cgcctcctggctcgagccggtgggatcgataaggttctttcatccattaaggctatgaag

R L L A R A G G I D K V L S S I K A M K

cctaagattgtcaccattgtggagcaagaagctaaccacaacggtccggtttttctagac

P K I V T I V E Q E A N H N G P V F L D

cggttcactgaagcgttgcattattactcgagtatgtttgactcgttagaaggatccggg

R F T E A L H Y Y S S M F D S L E G S G

atagcagttccgagtcaggacctggtcatgtccgagttgtacttaggaaggcagatttgc

I A V P S Q D L V M S E L Y L G R Q I C

aacgttgtggcttgtgaagggaccgacagagttgagcggcacgatattctgaaacattgg

N V V A C E G T D R V E R H D I L K H W

cgaactcggtttgaatcggctgggttcgatcctgttcatctcggttcgaacgcgtttaaa

R T R F E S A G F D P V H L G S N A F K

caagctggtatgctattagctctgtttgcaggtggtgatgggtacagagtggaggaaaat

Q A G M L L A L F A G G D G Y R V E E N

aatgggtgtcttatgcttgggtggcatactagaccggttatagctacctcggcgtggaaa

N G C L M L G W H T R P V I A T S A W K

ctcggcggttcacaatga

L G G S Q -tgggacatccttatctgctattcctgtatccacctggtaattttctgctgttttaattgaaatttgtatataactttatcacatgtaaaactaaaggaaaagccatattataagatgtagacatggttattaattacttaattattatagatttcaacccccc

***SsCYP707A2***

Accgcctgggtca atgggcttgccatatattggagagactcttcagctctactctcagaacccagattccttc

M G L P Y I G E T L Q L Y S Q N P D S F

tttgccaccaggcagaaaaggtatggagagatattcaagactcgcattcttgggtgccca

F A T R Q K R Y G E I F K T R I L G C P

tgtgtgatggtgagtagtcctgaggctatcaggtttgtgatggtgacacaggcaagcttg

C V M V S S P E A I R F V M V T Q A S L

ttcaagccaacctacccacctagcaaggagaaactgattggtccttcagcattatttttc

F K P T Y P P S K E K L I G P S A L F F

caccaagggagttaccatacacaaatgaggaagcttgtacaagcttctttctcccttgat

H Q G S Y H T Q M R K L V Q A S F S L D

gtaatcagaaacttggtacctcagattgaatctattgctatctctgtcttggattcatgt

V I R N L V P Q I E S I A I S V L D S C

tgtgttcaggctgttgttaaaactttccatcagatgaagaagttcacttttgatgctgct

C V Q A V V K T F H Q M K K F T F D A A

gttttatccatctttggtaatttggaacattgctatagagaaaagctgaagaaaaattac

V L S I F G N L E H C Y R E K L K K N Y

tgcatgctcgaccaaggttacaattctttcccaacatatctacctggatctttgttcagt

C M L D Q G Y N S F P T Y L P G S L F S

aactcagtcagggcaaggaaaaagataatcaagattctcaggcatataatatgggagagg

N S V R A R K K I I K I L R H I I W E R

aaggagaaaggtctattgcagaaagatcttttgggttgtctactaaatttcaaagatgat

K E K G L L Q K D L L G C L L N F K D D

aagggtgaaaccttgtctgatcatcaaattactgataacataatcggtgtattatttgct

K G E T L S D H Q I T D N I I G V L F A

gctcaggacaccacagctagtgtcttaacatggattcttaaatatattcatgatgactcc

A Q D T T A S V L T W I L K Y I H D D S

aaacttctagaagctattaagattgagcagatggcaatttttgaatcaaatgggaaaggg

K L L E A I K I E Q M A I F E S N G K G

aaaaatccattaacatggtctcaaactagaaatatgcccattacgaatcaggtaataatg

K N P L T W S Q T R N M P I T N Q V I M

gagagcctgaggatggcaagtgtcatatctttcacctttagggaagctgtagaagatgtt

E S L R M A S V I S F T F R E A V E D V

gaatataaaggatatttaattccaaaaggatggaaggtacttcctttgttcagaaacatt

E Y K G Y L I P K G W K V L P L F R N I

catcacaatccagatttcttcaatgatccacatgaatttaatccttcaagatttgagatt

H H N P D F F N D P H E F N P S R F E I

ggaccaaaacccaatacgtacatgccctttggtaatggtgttcatgcatgtcctggaaat

G P K P N T Y M P F G N G V H A C P G N

gaagtagccaagctagagatgatgattctaatccatcatctcgtcaccaagttcaggtgg

E V A K L E M M I L I H H L V T K F R W

gaagtcgtaggatcagtgggtagggttaaatatgatccatttccaatacccgaacaagga

E V V G S V G R V K Y D P F P I P E Q G

cttcctgctaagttctggaa

L P A K F W

***SsABI3***

aacggattatcaca

atgcttgacacggcgcagtcgtggcctgcgtcacaatatgctatggcttcatactatgcc

M L D T A Q S W P A S Q Y A M A S Y Y A

tcgtttggggacactaatcttcagcctgtgcagccccaccaggcagtttttactggctat

S F G D T N L Q P V Q P H Q A V F T G Y

ggaaatcaatatccatgtcagtatctacagggggaagttggtgacaggctggtgagatta

G N Q Y P C Q Y L Q G E V G D R L V R L

ggttcttctgctacaaaagaagcgaggaagaagagaatggcgagacagagaaggttttta

G S S A T K E A R K K R M A R Q R R F L

tcgcataacagaaatcagaatcagcagaataaccaacaaaaccagccccaaaatcagagt

S H N R N Q N Q Q N N Q Q N Q P Q N Q S

gctgaccaccatggaaggctaggaaatgataatggtgcaccaatagcggctcaagctaac

A D H H G R L G N D N G A P I A A Q A N

catgggaattgggtttattggtccacaaatcctgttcttcccgccgcatccatcccccag

H G N W V Y W S T N P V L P A A S I P Q

gttctacccatggatgcatcgccggtgcataggtttgaccggccggcggccatgcaaccg

V L P M D A S P V H R F D R P A A M Q P

gcccagaatcatcagccgcggcaagtggcatccgataggcgacagaattggaaatctgag

A Q N H Q P R Q V A S D R R Q N W K S E

aagaacctgaggtttcttctccagaaagtgttgaagcagagcgatgtgggtaatcttgga

K N L R F L L Q K V L K Q S D V G N L G

aggattgtgttgccaaaaaaagaagcagaaactcatcttccagaattagaggcaagagat

R I V L P K K E A E T H L P E L E A R D

ggcatttctattgcaatggaagatatagggacatctcgtgtttggaacatgcgttataga

G I S I A M E D I G T S R V W N M R Y R

ttctggcccaacaataaaagcaggatgtatctcctggaaaacacaggagattttgtgaga

F W P N N K S R M Y L L E N T G D F V R

acaaatgggctacaagaaggagatttcatagtcatctactcagacgtcaagtgtggcaaa

T N G L Q E G D F I V I Y S D V K C G K

tatttgataagaggagtgaaggtaaggctaccaggatcaaaattggagaacaataaagca

Y L I R G V K V R L P G S K L E N N K A

ggaaaatcacaaaagaatgcacatgcaagttcatcagctgctgggaacggtggttcttcg

G K S Q K N A H A S S S A A G N G G S S

tcactgatgcctgccactgtaaatcaaaatcaagctcaaactcaaacagtaaagtaa

S L M P A T V N Q N Q A Q T Q T V K -

tgagtagagagaaaaaagagagaatcaccaacaaacattctctctccttcatgcatgaaaatggatggacaagtggacggccgagattttctagggggtagttggtataaaaccctagattctctgcgtaatacagtgaccaatctcttgtttttcagtcaaaagcaatatagtttgttgcagatcacaactgccaaggtctattaagcaggagttggctctgggggcccatgcgttttattattgcctaatt

***SsGA3ox1***

ctcaactccataagcgtccactttataccatccaacctataaacgttttctccattgtagctacttctacttctactccaataacttccatttcccctacctaactaattacaggcccttttcctgtcaaaaatttcaacccactatattcttgtttaaacttattctcttccccactctatttttttctattaaaatattttgtcttctgatctctctattgtttccccttcttgtctacaaatttcact

atgccttcaaggctaacagacgcctttagagcccaccctgcccccgtcaatctccatcac

M P S R L T D A F R A H P A P V N L H H

aagcttcttgacttcacttctcttcaagaactgcctgactcctacaaatggactcaacta

K L L D F T S L Q E L P D S Y K W T Q L

gacgatcaccactcctccgccggcgacactatggaatcaattccggttatcgatcttttg

D D H H S S A G D T M E S I P V I D L L

gaccctaatgctcttaaaactataggatatgcatgcaaaaactggggtgtctttcaagtc

D P N A L K T I G Y A C K N W G V F Q V

attaaccatggcctcccttctagccttcttgataacgttgaggatgcttctaaaagtctc

I N H G L P S S L L D N V E D A S K S L

ttctctttacccgttcatcaaaaacttaaagctgaaagatcaccggacggtatttctggg

F S L P V H Q K L K A E R S P D G I S G

tatggtagagctagaatttcttccttcttctctaaacttatgtggtctgaaggattcacc

Y G R A R I S S F F S K L M W S E G F T

atcgttggatctccattggagcattttcgccacctctggcctcaagattgtactaaatac

I V G S P L E H F R H L W P Q D C T K Y

tgtgatataattgaagaataccagacagagatgcaaaagctagctggaagattgatgtgg

C D I I E E Y Q T E M Q K L A G R L M W

ctaatgttgggcccattagggataataagggaagacatcaaatgggccggcccaaaagct

L M L G P L G I I R E D I K W A G P K A

gatttcaaagaggcttctgcagctttacaaatgaattactacccggcttgcccggatccg

D F K E A S A A L Q M N Y Y P A C P D P

gatcgggccatgggtcttgctgcccataccgattcaactcttctcactatcctctaccag

D R A M G L A A H T D S T L L T I L Y Q

aacagtacaagtgggttgcaggttttaaaagaaggcaccgggtgggtaacagttccgcct

N S T S G L Q V L K E G T G W V T V P P

attccaggcgggcttgtaatcaatgtaggagatcttcttcacatattatcaaacgggtta

I P G G L V I N V G D L L H I L S N G L

tacccgagtgttcttcatcgggtattggttaaccggatcaaggatcgcctatccatggct

Y P S V L H R V L V N R I K D R L S M A

tatctgtatgggccaccgtcaagtgttcgaatatccccattatcaaaactagtaggccca

Y L Y G P P S S V R I S P L S K L V G P

actcaaccaccgctgtaccgccccgtcacttggaatgagtaccttggcactaaagctaag

T Q P P L Y R P V T W N E Y L G T K A K

cacttcaataaagcattatcgtcagttagggcctgtgctcctttaagcggattagttgat

H F N K A L S S V R A C A P L S G L V D

gtaaatgagcataataatagcgtccatgtaggctagcaaagccgaagaataccttct

V N E H N N S V H V G A

***SsGAI***

gttcctttatccttttccattttcactcctctgtttctattcttcctccattcaagctcacataaatttgaaactgaagcataattccaataaaattgatgctcgtattactgtgaatcgagaagagagaaaaaaaaag

atgaagagagatcatcaagaaagtgctggtggttgtggtgcgggcaaaggggagccctct

M K R D H Q E S A G G C G A G K G E P S

tctatggcgagaggaaaaatttgggaagaagaacaagatggtgcaggtatggatgaattg

S M A R G K I W E E E Q D G A G M D E L

cttgctgtcttgggttacaaggttcgatcttctgatatggcagatgtagctcagaagctt

L A V L G Y K V R S S D M A D V A Q K L

gaacagcttgaaatggtgatgggtactgctcaagaagatgggatttcacagctttctgat

E Q L E M V M G T A Q E D G I S Q L S D

accgtacattataatccctctgatctctctggttgggttcagagtatgctctctgagctt

T V H Y N P S D L S G W V Q S M L S E L

aatccagccactggacttgatgattctgtttttggttcttctgaaattagtttctctaat

N P A T G L D D S V F G S S E I S F S N

cctcaatcgcaccaatcgggtcttttcaatgatgattcggaatatgatctccgagcaatt

P Q S H Q S G L F N D D S E Y D L R A I

cccggtgtggtgggttacccacctgaggaaaatagcaggaagaaaatgaaaacctctatg

P G V V G Y P P E E N S R K K M K T S M

gggtctgatacagctgattcgaatccgaaaactccttcattagcttcaacttctggctcg

G S D T A D S N P K T P S L A S T S G S

ccgtcgcgagcggtggtgctaatcgactcgcaggagaccggagtccgcctagtgcatacg

P S R A V V L I D S Q E T G V R L V H T

ttattagcttgtgctgaagcagttcagcaagacaatttaaagctcgcagaggcactcgtc

L L A C A E A V Q Q D N L K L A E A L V

aaacacattggcttacttgctgcatctcaagccagtgctatgaggaaagtggcgacttac

K H I G L L A A S Q A S A M R K V A T Y

ttcgccgaagctttagctcgtcgaatttacaaaatctaccctcaagaatccctcctcgat

F A E A L A R R I Y K I Y P Q E S L L D

ccttcttattccgatactttagagatgcatttttacgagacttgcccttatctcaaattc

P S Y S D T L E M H F Y E T C P Y L K F

gcccattttactgctaatcaagccattcttgaagcttttggtactgcaaacagagttcat

A H F T A N Q A I L E A F G T A N R V H

gtcattgatttcggtctcaaacagggaatgcagtggccggccctaatgcaagctctcgct

V I D F G L K Q G M Q W P A L M Q A L A

ttgcgtcctggtggtcctcccactttccggttaaccggaatcggccctccgcagcccaat

L R P G G P P T F R L T G I G P P Q P N

aacaccgatactttgcagcaggtggggtggaagctagctcagttagctgaaactattggc

N T D T L Q Q V G W K L A Q L A E T I G

gtggaattcgaattccgcggtttcgtagctaatagtttagcagatcttcaaccggaaatt

V E F E F R G F V A N S L A D L Q P E I

ctggacctccgctccccggaggtggagacggtggttgtcaactccgttttcgagctccac

L D L R S P E V E T V V V N S V F E L H

cgcctcctggctcgagccggtgggatcgataaggttctttcatccattaaggctatgaag

R L L A R A G G I D K V L S S I K A M K

cctaagattgtcaccattgtggagcaagaagctaaccacaacggtccggtttttctagac

P K I V T I V E Q E A N H N G P V F L D

cggttcactgaagcgttgcattattactcgagtatgtttgactcgttagaaggatccggg

R F T E A L H Y Y S S M F D S L E G S G

atagcagttccgagtcaggacctggtcatgtccgagttgtacttaggaaggcagatttgc

I A V P S Q D L V M S E L Y L G R Q I C

aacgttgtggcttgtgaagggaccgacagagttgagcggcacgatattctgaaacattgg

N V V A C E G T D R V E R H D I L K H W

cgaactcggtttgaatcggctgggttcgatcctgttcatctcggttcgaacgcgtttaaa

R T R F E S A G F D P V H L G S N A F K

caagctggtatgctattagctctgtttgcaggtggtgatgggtacagagtggaggaaaat

Q A G M L L A L F A G G D G Y R V E E N

aatgggtgtcttatgcttgggtggcatactagaccggttatagctacctcggcgtggaaa

N G C L M L G W H T R P V I A T S A W K

ctcggcggttcacaatga

L G G S Q gtcaattcatctgagttactgagttggaattaacgagtcagtgaatcttgagatatcttagtgagatgagggagtaaaaagcaattcaagtgtgtaatttctctagtctttttaacacctcttgcttattttattttacataagtttttttttcttttttacttgctgtaatcttagaatttttat

***SsGA2OX***

atgaggctcaactattatcctccatgccaaaaacctgaccttactttaggaacagggcct

M R L N Y Y P P C Q K P D L T L G T G P

cattgtgatccaacttcattaacaatccttcatcaggaccaagtgggcggtcttcaagtc

H C D P T S L T I L H Q D Q V G G L Q V

tttgtagacgatgaatggcgctcaattacccctaacttcgatgcttttgtcgttaacatt

F V D D E W R S I T P N F D A F V V N I

ggcgacactttcatggcactttcaaatgggaaatacaagagttgcttgcatagagcagta

G D T F M A L S N G K Y K S C L H R A V

gtgaacagcgaaacaccaaggaaatcactggctttctttctgtgtccaaagagtgacaaa

V N S E T P R K S L A F F L C P K S D K

atcgtaaaaccaccaacacaactagtggacacttataatccaagaatatatccagacttt

I V K P P T Q L V D T Y N P R I Y P D F

acatggccaatgctcctcgaatttactcagaagcattacagagctgacatgaagactctt

T W P M L L E F T Q K H Y R A D M K T L

gaaatgttcacaaattgggttcaagttcaacagagaaagtag

E M F T N W V Q V Q Q R K -

***SsRGL2***

gttcctttatccttttccattttcactcctctgtttctattcttcctccattcaagctcacataaatttgaaactgaagcataattccaataaaattgatgctcgtattactgtgaatcgagaagagagaaaaaaaaag

atgaagagagatcatcaagaaagtgctggtggttgtggtgcgggcaaaggggagccctct

M K R D H Q E S A G G C G A G K G E P S

tctatggcgagaggaaaaatttgggaagaagaacaagatggtgcaggtatggatgaattg

S M A R G K I W E E E Q D G A G M D E L

cttgctgtcttgggttacaaggttcgatcttctgatatggcagatgtagctcagaagctt

L A V L G Y K V R S S D M A D V A Q K L

gaacagcttgaaatggtgatgggtactgctcaagaagatgggatttcacagctttctgat

E Q L E M V M G T A Q E D G I S Q L S D

accgtacattataatccctctgatctctctggttgggttcagagtatgctctctgagctt

T V H Y N P S D L S G W V Q S M L S E L

aatccagccactggacttgatgattctgtttttggttcttctgaaattagtttctctaat

N P A T G L D D S V F G S S E I S F S N

cctcaatcgcaccaatcgggtcttttcaatgatgattcggaatatgatctccgagcaatt

P Q S H Q S G L F N D D S E Y D L R A I

cccggtgtggtgggttacccacctgaggaaaatagcaggaagaaaatgaaaacctctatg

P G V V G Y P P E E N S R K K M K T S M

gggtctgatacagctgattcgaatccgaaaactccttcattagcttcaacttctggctcg

G S D T A D S N P K T P S L A S T S G S

ccgtcgcgagcggtggtgctaatcgactcgcaggagaccggagtccgcctagtgcatacg

P S R A V V L I D S Q E T G V R L V H T

ttattagcttgtgctgaagcagttcagcaagacaatttaaagctcgcagaggcactcgtc

L L A C A E A V Q Q D N L K L A E A L V

aaacacattggcttacttgctgcatctcaagccagtgctatgaggaaagtggcgacttac

K H I G L L A A S Q A S A M R K V A T Y

ttcgccgaagctttagctcgtcgaatttacaaaatctaccctcaagaatccctcctcgat

F A E A L A R R I Y K I Y P Q E S L L D

ccttcttattccgatactttagagatgcatttttacgagacttgcccttatctcaaattc

P S Y S D T L E M H F Y E T C P Y L K F

gcccattttactgctaatcaagccattcttgaagcttttggtactgcaaacagagttcat

A H F T A N Q A I L E A F G T A N R V H

gtcattgatttcggtctcaaacagggaatgcagtggccggccctaatgcaagctctcgct

V I D F G L K Q G M Q W P A L M Q A L A

ttgcgtcctggtggtcctcccactttccggttaaccggaatcggccctccgcagcccaat

L R P G G P P T F R L T G I G P P Q P N

aacaccgatactttgcagcaggtggggtggaagctagctcagttagctgaaactattggc

N T D T L Q Q V G W K L A Q L A E T I G

gtggaattcgaattccgcggtttcgtagctaatagtttagcagatcttcaaccggaaatt

V E F E F R G F V A N S L A D L Q P E I

ctggacctccgctccccggaggtggagacggtggttgtcaactccgttttcgagctccac

L D L R S P E V E T V V V N S V F E L H

cgcctcctggctcgagccggtgggatcgataaggttctttcatccattaaggctatgaag

R L L A R A G G I D K V L S S I K A M K

cctaagattgtcaccattgtggagcaagaagctaaccacaacggtccggtttttctagac

P K I V T I V E Q E A N H N G P V F L D

cggttcactgaagcgttgcattattactcgagtatgtttgactcgttagaaggatccggg

R F T E A L H Y Y S S M F D S L E G S G

atagcagttccgagtcaggacctggtcatgtccgagttgtacttaggaaggcagatttgc

I A V P S Q D L V M S E L Y L G R Q I C

aacgttgtggcttgtgaagggaccgacagagttgagcggcacgatattctgaaacattgg

N V V A C E G T D R V E R H D I L K H W

cgaactcggtttgaatcggctgggttcgatcctgttcatctcggttcgaacgcgtttaaa

R T R F E S A G F D P V H L G S N A F K

caagctggtatgctattagctctgtttgcaggtggtgatgggtacagagtggaggaaaat

Q A G M L L A L F A G G D G Y R V E E N

aatgggtgtcttatgcttgggtggcatactagaccggttatagctacctcggcgtggaaa

N G C L M L G W H T R P V I A T S A W K

ctcggcggttcacaatga

L G G S Q - gtcaattcatctgagttactgagttggaattaacgagtcagtgaatcttgagatatcttagtgagatgagggagtaaaaagcaattcaagtgtgtaatttctctagtctttttaacacctcttgcttattttattttacataagtttttttttcttttttacttgctgtaatcttagaatttttat

***SsMPK6***

aaaaaaaaaaataccaaacagaaaagcagagagaaacagagcttcagatcacaacacgtatatatgaaagacaagaccccatagttttatagcttcagatctgtttgacccaaatttcccaactaatttttcagaa

atggacggggttggagcagcccagccggcagacactgagatggcggaagcgtcgaccacg

M D G V G A A Q P A D T E M A E A S T T

gccgcacccccaccttctgagtcccaacaacaggttcctcctaccggcggcatcgagact

A A P P P S E S Q Q Q V P P T G G I E T

attccggcctcccttagccatggaggcaggttcattcagtacaacatattcggtaacata

I P A S L S H G G R F I Q Y N I F G N I

tttgaggtcactgctaaatataagcctcctatcttgcccatcggcaagggtgcttacggc

F E V T A K Y K P P I L P I G K G A Y G

atcgtttgctcggcattgaattcggagaccggagagcatgtggcaatcaagaagatagcc

I V C S A L N S E T G E H V A I K K I A

aatgcttttgataataaaatcgacgctaaaaggaccctccgcgagatcaagttgcttcga

N A F D N K I D A K R T L R E I K L L R

catatggatcatgaaaacgttgttgcaatcagggatataatgcctccaccccaaagggaa

H M D H E N V V A I R D I M P P P Q R E

gcatttaatgatgtttacatcgcatatgaacttatggacactgatctgcatcagattatt

A F N D V Y I A Y E L M D T D L H Q I I

cgttccaatcaagcattatcagaagagcactgccagtattttctatatcaaattctccga

R S N Q A L S E E H C Q Y F L Y Q I L R

gggttgaaatatattcattctgctaatgttctgcatagagatttaaaaccaagcaatctc

G L K Y I H S A N V L H R D L K P S N L

ctcctgaacgcaaattgtgacctaaagatttgtgattttggtctagctcgtgttacatca

L L N A N C D L K I C D F G L A R V T S

gaaactgatttcatgacagaatacgttgttacaagatggtatcgagcaccagaattgctg

E T D F M T E Y V V T R W Y R A P E L L

ttgaactcttctgactatactgcagctattgatgtatggtcagtgggttgtattttcatg

L N S S D Y T A A I D V W S V G C I F M

gagttgatggatcgaaggcccttatttcctggcagagatcatgtgcatcagcttcgtttg

E L M D R R P L F P G R D H V H Q L R L

cttatggagctaatcggcactccctcagaggctgaattaggatttttgaatgaaaatgca

L M E L I G T P S E A E L G F L N E N A

aagagatacattcgacaacttcccgtttatcgtcggcaatctttcactgagaagttccca

K R Y I R Q L P V Y R R Q S F T E K F P

aatgttcacccagcagctattgatcttgttgaaaagatgttgacatttgacccaagacaa

N V H P A A I D L V E K M L T F D P R Q

aggattacagttgaggatgcactggctcatccctacctaacatcactgcacgacattagt

R I T V E D A L A H P Y L T S L H D I S

gacgagccagtgtgcatgactcccttcagctttgattttgagcagcatgctttgactgaa

D E P V C M T P F S F D F E Q H A L T E

gaacagatgaaggaactgatttatagggaggcacttgcatttaaccctgagtatcaacat

E Q M K E L I Y R E A L A F N P E Y Q H

caatga

Q -

Taatacaagcatcagtgggtataaattcattgtggctttgatcagtgtgatttccgttagttgaatgatttcagtaatttatttcatgtatataagttccaccaaactgggtggttcttcaactcgagtatttgcttatgggctgaaccggggaacgaaaatatttgaattagtgtttgtatcggctattttgcttattgttgggctcttaatttaatccattctctaagacaggaaatttattctctctctaagatacaggaaattaggcaaatgatc

***SsNLP8***

atttttttttaggtcttgtttctctattttccttagggttagggttttcgattggggatttctgtatttggtgtctttaccaatttagcgtccattttcagctgaatttaagattgtttttctgtaaagtctgtgtgttcgatacccagatggaatatgtatggataatggatatggatacgtgtatttttgagtgcgctacaggacaaattgaagatggatttcgtgagttgttattgattgaaaagtgtggacttacttgcgccagctttggagattgtgaattgttgggcgtcatctcttagaggccagttttggagatttcctttctgtattaaggaatgggattggaaagaaatcagattttgctctgcttctagtcttgtgttactccaatttatccagtggaagtttgggtctttagtggattcctgtggtagctaagataaatgtctgagcattaagggtataacttttttaagattttagtatgaaatttagtagtttcacaaacaaatgagagaagatattaggggagactttctttgaatagagaagtaaatttctttacataggttgggaacttgggggaatttgtgggttgataaactaga

atggaataccctttctcatcaaaggaaaaggggatcaattattggacgtcatcaccagca

M E Y P F S S K E K G I N Y W T S S P A

caaacagatggtatggctttaccaggtggtggtacgaggaatccaatttcagaagaccta

Q T D G M A L P G G G T R N P I S E D L

ttcaacaatttttcagagctcatgaattttgatacatatgccggttggtgcaacagtcca

F N N F S E L M N F D T Y A G W C N S P

gcagcaactgatcagatgtgtgctgcctatgggttgccatcttttcattcaacaccctat

A A T D Q M C A A Y G L P S F H S T P Y

gcatcttttgaagcattgaatttttcagaacagaattgttcaaccattcctgatggggac

A S F E A L N F S E Q N C S T I P D G D

aagtcattagatgctgctggtccatctagctgctatggagacaaaatggaatttcggcga

K S L D A A G P S S C Y G D K M E F R R

gattcccaattcgcttatccatcagattcagtgcatgctgatgatatggttgaaaagcaa

D S Q F A Y P S D S V H A D D M V E K Q

agtaacaatgctcagcaacaaagaattatgtctaatatggcaaattgtgttatttctaga

S N N A Q Q Q R I M S N M A N C V I S R

ccacttgggttttcacttgatgaaaagatgctgagggcattatcgatgttaaaagattct

P L G F S L D E K M L R A L S M L K D S

gctggtgggggcattttggcacaagtttgggtcccaatgaggcgtggggatcaatatatc

A G G G I L A Q V W V P M R R G D Q Y I

atgaccacttatgagcaaccttacttgctcgatcaaagtctggcaggctatcgtgaagtg

M T T Y E Q P Y L L D Q S L A G Y R E V

tcaaggacatataccttctccgcagaaggcaagtctggccttcctcttgggcttcctggt

S R T Y T F S A E G K S G L P L G L P G

cgtgtatttatctttaaagttccagagtggacttcaaatgtagtatattatagcaatgct

R V F I F K V P E W T S N V V Y Y S N A

gaatacttaagagtaaagcatgcacttcatcataaagtccgaggttctattgcgttgcca

E Y L R V K H A L H H K V R G S I A L P

atttttgaacctcctcacatgtcctgctgtgctgtacttgaacttgtcacagttagagag

I F E P P H M S C C A V L E L V T V R E

aagcccgattttgattcagagatagaaaatgtttgccatgcactccaggctgtaaattta

K P D F D S E I E N V C H A L Q A V N L

aggagcacaggacctcctcgacttcttactcagagcctctcaaggaataaagtggctgcc

R S T G P P R L L T Q S L S R N K V A A

ttggctgaaataacagacgttttacgagtcgtatgtcatgcacatagattgccactggct

L A E I T D V L R V V C H A H R L P L A

ttaacatggattccttgcaattttgtggaagaagctgttgatgaaattataaaagtacgt

L T W I P C N F V E E A V D E I I K V R

gtcagagaaggtaatttaaggtctacagggaaatctgtgctatgcattcagggtacagct

V R E G N L R S T G K S V L C I Q G T A

tgttatgcaagtagatatatggaaggatttgtccatgcatgttcagaaaattatattgag

C Y A S R Y M E G F V H A C S E N Y I E

gaacgacagggtgtagctgggaaagctcttcaatcaaatcatcccttcttcttccctgat

E R Q G V A G K A L Q S N H P F F F P D

gtgaagtcatatgatataaccgagtatccactagtccatcatgcacgcaagtatagcttg

V K S Y D I T E Y P L V H H A R K Y S L

aatgctgctgttgcaatcaggctaagaagcacttacactggtgatgatgattacatatta

N A A V A I R L R S T Y T G D D D Y I L

gagttctttctccctatcaatatgaaggggagctcagaacaggaacttttgttgaacaac

E F F L P I N M K G S S E Q E L L L N N

ctctcaggcaccatgcagaaggtttgtaagagtttgagaacagtttcagatgcagagtta

L S G T M Q K V C K S L R T V S D A E L

ggaggtggagaaggttctgtagttgagtttcagaaagcaactatgtccagcttcccacag

G G G E G S V V E F Q K A T M S S F P Q

ttgtcggtttcagtaataaactcgaaagcagcactattaaatgccaacttgaatttggtt

L S V S V I N S K A A L L N A N L N L V

gacaaggtacctttagatgtatctagttcaaaaaatgatgtgacagaatcaaatggtcct

D K V P L D V S S S K N D V T E S N G P

tgtgaacaggcaatgagtggaccgagaagacagctggagaagaagaggagtacagcagag

C E Q A M S G P R R Q L E K K R S T A E

aaaaatgtgagcttggatgttcttcagcaatatttttctggaagtctcaaggatgctgcc

K N V S L D V L Q Q Y F S G S L K D A A

aaaagcattggcgtttgccccacaacactgaaaaggatatgcagacaacatgggatctct

K S I G V C P T T L K R I C R Q H G I S

agatggccatccagaaagataaataaggtgaatcgctcattaaggaaaatacagactgtg

R W P S R K I N K V N R S L R K I Q T V

cttgactctgttcagggagtggaaggaggactgatatttgatccgaccactgtgggattt

L D S V Q G V E G G L I F D P T T V G F

gtggcagcaagctccataatccaagaacatgataatcgaaaaaactttctatttcctgac

V A A S S I I Q E H D N R K N F L F P D

aaaaactttcctgctagaaattatgaatctgcctctgaagatgctgtttctgtacctcca

K N F P A R N Y E S A S E D A V S V P P

gctccttgtaatgatggcaataattctacagttaaagtcgaagaggatgactgctgtgtg

A P C N D G N N S T V K V E E D D C C V

gatgtctatgggggttttatgaaagctagcattcccatggttgattgtagtgaagaatcc

D V Y G G F M K A S I P M V D C S E E S

aagtcttttgcaacggaagctggactagtccagaaagctagcgttggctctggaccttgg

K S F A T E A G L V Q K A S V G S G P W

gcttatcgggaaaatccctctacagttacaaaaggaggctactggtgtctgaacaaggat

A Y R E N P S T V T K G G Y W C L N K D

agcttgaaattggagaatttgaatgcccattttatgtctcgaagttcatgttccttgtct

S L K L E N L N A H F M S R S S C S L S

gttgctaatgaattggattccaaaatgcagattgatgatgaaacattggaacataatcag

V A N E L D S K M Q I D D E T L E H N Q

cctacttgttcaagcatgacagactcttcaaatggctctggttcattgattcatggcagt

P T C S S M T D S S N G S G S L I H G S

acatcaagctcactgagctttgaggagggaaagaatttaaaagtgaaaacaagctgtgat

T S S S L S F E E G K N L K V K T S C D

gatagtggatcaaaaattattgtaaaagccacgtataaagaagacaaagttaggttcaag

D S G S K I I V K A T Y K E D K V R F K

tttgaaccatctgcagggtgtttccaattgtatgaagaagttgcgaaaaggttcaaattg

F E P S A G C F Q L Y E E V A K R F K L

caatatggaacattccagctcaagtatcttgatgatgaagatgagtgggtgatgcttgtg

Q Y G T F Q L K Y L D D E D E W V M L V

agcgactccgacttgcaggagtgtgttgacatcttggatcttgttgggaatcgaactgtg

S D S D L Q E C V D I L D L V G N R T V

aaattccttgttcgtgatacgcctacttctatgggcagctcaggcagtagcaattgcttt

K F L V R D T P T S M G S S G S S N C F

ttgggaggaagctcttag

L G G S S -

Agcttgaggtgatgctcaatgcatgggctgttactcgttcatgttgtttttgcaattttttgtggcttctatttcatgaaagttgtattagtactatgaaggttaaacagggagtgtggaatggccataacttctctgcagcggttctttttttcaaaatttttgctgaaccaatggcagagtgaggcatcgcctttcttcgggctgaacataccacagttcagaactacgagatgaacagtccaaagtgcagctgagtggtgagatttcctgggatggaagaaaggggtggaattgaaaaggcgagaatatatgtttggtagcacattagatgttaactgtatatgtttgtatattgtaggttgtaatgagaagttactatagctgtagaaacgctcgaaggcactatatccaatgttatccagtgccttagtctcaatctgtacagtaattt

***SsCIPK23***

cttagatttcattggtaattgcaatcctcccatattcatataaaaaggccttagatttcatttcgaatttactctccaacccttgtcacgcacatatttctattttctatctcaaaagctttccactttctgtaacgaattttgtttttccaactggaaacaaccaccaagatccacagctactcgcaccgttttcagcttcctcttatactttctgaaggcgaaatatagccttttattttcctgagaaaaaaaaaattgaaaatcagctccgttaaattgtccggctttatgggtttattgtctgattcaaccgcaaatatgtttttctctctctctttggagacagtttccttaattttagagatttaaatttgcttccttcttggtatagactttagagctttccttgttctctataattagtttggttctttttccagtgtttgtattaattcgaaggaaggaaaaaacaaaaaggacgatgagtgtggcgaagtctcaagtgtggcaaccgtgtaaaaagaagagatcttgacggattaggaacatagaggcagagagggagttaaaacttggaattttttaagggaaatttggaatcctaaagagggcggaagagggagaggagaa

atggcttcacgagggagcgctgcgtctaacagtaggactcgcgtgggaaggtacgagctt

M A S R G S A A S N S R T R V G R Y E L

ggaaggacgctgggagaaggaacatttgcgaaagtgaagtttgctaggaatatcgagacc

G R T L G E G T F A K V K F A R N I E T

ggagagaatgtcgccattaagattctcgataaggagaaggttcttaagcataaaatgatc

G E N V A I K I L D K E K V L K H K M I

gttcagattaaacgtgaaatatcaaccatgaaattgatcagacacccaaacgtgatccgt

V Q I K R E I S T M K L I R H P N V I R

atgtatgaggtgatggcaagcaagtcaaaaatatatattgttctggaatttgttactggt

M Y E V M A S K S K I Y I V L E F V T G

ggtgaactttttgacaaaattgcaagcagagggaggttgaaagaggatgaagcaaggaag

G E L F D K I A S R G R L K E D E A R K

tacttccagcagcttgtcaatgctgtagattactgtcatagcagaggtgtataccataga

Y F Q Q L V N A V D Y C H S R G V Y H R

gacctaaagcctgagaatctgctacttgatgctagtggagtacttaaagtttcagatttt

D L K P E N L L L D A S G V L K V S D F

ggattaagtgcactaccgcaacaagttcgagaagatggattactacacacaacatgtgga

G L S A L P Q Q V R E D G L L H T T C G

acgccaaactatgttgccccagaggtcataaacaataaaggctatgatggtgctaaagca

T P N Y V A P E V I N N K G Y D G A K A

gatttatggtcatgtggcgtgattctttttgttttaatggctggttacttgccttttgaa

D L W S C G V I L F V L M A G Y L P F E

gaacctaaccttatgactctttacaagaagatatttaaggcagatttcacatgtcctcca

E P N L M T L Y K K I F K A D F T C P P

tggttctcctcaagtgcaaagaaattaattaaaagaattctagatcctaatcctttgaca

W F S S S A K K L I K R I L D P N P L T

cgtattaccatcgcggaagttattgaaaatgagtggtttaagaaagggtataagccgcct

R I T I A E V I E N E W F K K G Y K P P

acttttgaacaagctgaagttagtcttgatgatgtgaactctatcttcagtgaatctggg

T F E Q A E V S L D D V N S I F S E S G

gattgtcgggaccttgttgtggagaggcgagaaactcctatagggccagtggcacctata

D C R D L V V E R R E T P I G P V A P I

actatgaatgcatttgaacttatctctacatctcagggtctcaacctcagtagtcttttt

T M N A F E L I S T S Q G L N L S S L F

gagaaacaaatggggcttgtgaaacgagaaacaagatttacatccaaacattctgctaat

E K Q M G L V K R E T R F T S K H S A N

gagataatctcaaaaattgaagcagctgcagggcctttgggttttgaagtaaagaaaaat

E I I S K I E A A A G P L G F E V K K N

aattttaagatgaagcttcaaggggagaagactggacgcaaaggtcatttatctattgca

N F K M K L Q G E K T G R K G H L S I A

acggaaatatttgaggtggccccttctctttatatggttgagcttcgcaaatctggtgga

T E I F E V A P S L Y M V E L R K S G G

gacactctagaatttcacaagttctacaagaacctgtcggctggactaaaagatattgtt

D T L E F H K F Y K N L S A G L K D I V

tggaaaactgttgatgaagagaaggaagaggaggtggagagaaatggtgctggtgtgctt

W K T V D E E K E E E V E R N G A G V L

cggccataa

R P -

cagttggcttcttttgatggtccgagttgtttagtggctcttttcttcccagaaaagtccaaacatgcgattatccaaaaaaaaaacatgttatctaaagcttactgtgtttatattaattgttatggaagggattgagaatgcattgtattgtgattttcttaagaattgagtttaatgatcaagaacttgatcatgtatataatttggttttctgaaaatatgaatgcatcataacttttctcccgtctttcttctaggcaggagaaaacatattttaatctgatttcgctgtttccctttttttttttt
