# Supplementary material for: Proanthocyanidins in seed coat tegmen and endospermic cap inhibit seed germination in Sapium sebiferum
Source: PeerJ. 2018 Apr 26;6:e4690. doi: 10.7717/peerj.4690 (PMC5924686; doi:10.7717/peerj.4690)
Supplement: Supplemental Information 9 — The CDS of each gene was used to design primers. All the primers for qPCR were designed by using Primer Premier 6.0. [file peerj-06-4690-s009.docx]

| **Gene Name** | **Primer Type** | **Sequence( 3'------5' end)** |
| --- | --- | --- |
| *SsDOG1* | Sense Primer | CCCACAACGATGCTTCAGCCTATT |
|  | Anti-sense Primer | AGCCACAGAGTGCGTATAAGAGTCT |
| *SsNCED6* | Sense Primer | CGGAGAATCAACTCGGACGGTTATAG |
|  | Anti-sense Primer | CCACGGTTCTGCTATTGCCATGT |
| *SsCYP707A2* | Sense Primer | AGACTCTTCAGCTCTACTCTCAGAACC |
|  | Anti-sense Primer | GCCTCAGGACTACTCACCATCACA |
| *SsABI3* | Sense Primer | AAGAGAATGGCGAGACAGAGAAGGT |
|  | Anti-sense Primer | TGCGGCGGGAAGAACAGGAT |
| *SsGA2OX* | Sense Primer | CAAGTGGGCGGTCTTCAAGTCTTT |
|  | Anti-sense Primer | TTCGCTGTTCACTACTGCTCTATGC |
| *SsGAI* | Sense Primer | CGCAGCCCAATAACACCGATACTT |
|  | Anti-sense Primer | TGACAACCACCGTCTCCACCTC |
| *SsGA3OX1* | Sense Primer | GCTGGAAGATTGATGTGGCTAATGTTG |
|  | Anti-sense Primer | TCTGGTAGAGGATAGTGAGAAGAGTTGAAT |
| *SsRGL2* | Sense Primer | CGCAGCCCAATAACACCGATACTT |
|  | Anti-sense Primer | TGACAACCACCGTCTCCACCTC |
| *SsMPK6* | Sense Primer | GCAGGTTCATTCAGTACAACATATTCGG |
|  | Anti-sense Primer | TCTTCTTGATTGCCACATGCTCTCC |
| *SsNLP8* | Sense Primer | TCTGGCAGGCTATCGTGAAGTGT |
|  | Anti-sense Primer | GCAACGCAATAGAACCTCGGACTT |
| *SsCIPK23* | Sense Primer | TGTATGAGGTGATGGCAAGCAAGTC |
|  | Anti-sense Primer | GCTATGACAGTAATCTACAGCATTGACAAG |
| *SsACTIN* | Sense Primer | GTATCGTGTTGGATTCTGGTGATGGT |
|  | Anti-sense Primer | CGGCAGTGGTGGTGAAGGAGTA |
